# Characterization of the Resistance of SJL/J Mice to Pneumonia Virus of Mice, a Model for Infantile Bronchiolitis Due to a Respiratory Syncytial Virus

**DOI:** 10.1371/journal.pone.0044581

**Published:** 2012-10-15

**Authors:** Stephanie Glineur, Dao Bui Tran Anh, Michaël Sarlet, Charles Michaux, Daniel Desmecht

**Affiliations:** Department of Morphology and Pathology, College of Veterinary Medicine, University of Liege, Sart Tilman, Belgium; University of Iowa, United States of America

## Abstract

Respiratory syncytial virus (RSV), a prominent cause of airway morbidity in children, maintains an excessive hospitalization rate despite decades of research. Host factors are assumed to influence the disease severity. As a first step toward identifying the underlying resistance mechanisms, we recently showed that inbred mouse strains differ dramatically as regards their susceptibility to pneumonia virus of mice (PVM), the murine counterpart of RSV. PVM infection in mice has been shown to faithfully mimic the severe RSV disease in human infants. This study aimed at dissecting the remarkable PVM-resistance shown by the SJL/J strain. To characterize its genetic component, we assessed clinical, physiopathological, and virological resistance/susceptibility traits in large first (F1) and second (F2) generations obtained by crossing the SJL/J (resistant) and 129/Sv (susceptible) strains. Then, to acquire conclusive *in vivo* evidence in support of the hypothesis that certain radiosensitive hematopoietic cells might play a significant role in PVM-resistance, we monitored the same resistance/susceptibility traits in mock- and γ-irradiated SJL/J mice. Segregation analysis showed that (i) PVM-resistance is polygenic, (ii) the resistance alleles are recessive, and (iii) all resistance-encoding alleles are concentrated in SJL/J. Furthermore, there was no alteration of SJL/J PVM-resistance after immunosuppression by γ-irradiation, which suggests that adaptive immunity is not involved. We conclude that host resistance to pneumoviruses should be amenable to genetic dissection in this mouse model and that radioresistant lung epithelial cells and/or alveolar macrophages may control the clinical severity of pneumovirus-associated lung disease.

## Introduction

The respiratory syncytial virus (RSV) is a major pathogen of the human species. By the age of 18 months, about 85% of all infants are already seropositive, and practically all children are seropositive by the time they reach two years of age [1]. Even though the viral disease associated with infection is most often benign, the proportion of severe, life-threatening clinical cases is surprisingly high as compared to other respiratory viruses. Statistics show that each winter, in developed countries, about 2.3% of the children born in the year are hospitalized for severe respiratory symptoms caused by RSV infection [2]. This virus is also estimated to be responsible for 3 to 9% of the mortality of children fewer than five years of age suffering from diseases of the lower airways [3]. Traditionally, the risk factors underlying clinically severe cases of RSV disease include prematurity, chronic lung disease, congenital heart disease and immunodeficiency [4]. As the great majority of children hospitalized do not belong to any of these categories [5], it has been suggested that individual factors, including genetic ones, might influence the clinical severity of the viral disease associated with RSV infection [6]. In fact, the possibility of a genetic vulnerability is supported by a recent study which showed an increased concordance of severe RSV infection in monozygotic twins over dizygotic twins and which evaluated heritable contribution to the disease at ∼20% [7]. The genetic determinism of resistance/susceptibility to an infectious disease is often complex, making it hard to establish a causal relationship between clinical severity and any one gene [8]. The classic strategy is to conduct population-based association studies aiming to demonstrate that certain specific allelic variants are more frequent in hospitalized children than in children with few or no symptoms [9]. Such studies have demonstrated that specific haplotypes at the IL4 [10], [11], [12], [13], IL8 [14], [15], [16], IL9 [17], IL10 [18], [19], IL18 [20], CCR5 [21], CCL5 [22], CX3CR1 [23], IGHG2 [24], TLR4 [25], [26], [27], SP-A [28], [29], [30], SP-B [31], SP-C [32] and SP-D [33] loci are associated with the severe clinical form. Despite these successes, the large number of candidate genes raises questions, and obtaining robust replication of population-based association study findings has proven very difficult [34].

Alternatively, complex resistance/susceptibility traits can be dissected in genetically well-defined inbred strains of mice, where particular genes may have been randomly fixed. This approach seems particularly legitimate in the present case, because a virus is available that is both well adapted to mouse and phylogenetically very close to RSV [35]. What's more, this virus faithfully reproduces the human disease [36], [37]: (i) the clinical picture found in mice consistently mimics that observed in infants with RSV-associated disease, (ii) the dramatic granulocytic infiltrations observed in mouse parallel the pathological changes observed in human lungs, (iii) there is clear evidence of widespread viral replication in lung tissue, with incremental recoveries at peak in excess of 10^8^ plaque-forming units (PFU) per gram in response to as few as 30 PFUs in the inoculum, and (iv) there is a clear progression to ARDS, as reported for ∼3% of infants with RSV disease [38], [39]. Using this model in a screen of six inbred mouse lines, we recently revealed a pattern of continuous variation of resistance/susceptibility to PVM, with resistant (SJL/J), intermediate (BALB/c, C57BL/6, C3H-HeN, DBA/2), and susceptible (129/Sv) strains [40]. In the present study, our aim was to dissect further the PVM-resistance genotype and phenotype. To characterize the genetic component underlying the mouse response to PVM, we assessed clinical, physiopathological, and virological resistance/susceptibility traits in large first (F1) and second (F2) generations derived from the SJL/J and 129/Sv strains. Then, to test conclusively *in vivo* whether a functioning adaptive immune system is critical for surviving a PVM infection, we monitored the same resistance/susceptibility traits in mock- and γ-irradiated SJL/J mice.

## Materials and Methods

### Design

Three hundred and forty mice were enrolled in the segregation study aiming to measure the heritability of different PVM resistance traits and to characterize their most likely genetic determinism. This cohort included 25 mice of each parental strain, 43 F1 hybrids, and 247 F2 descendants. The live weight and plethysmographic respiratory parameters of each mouse were measured before virus inoculation and daily for 7 days post-inoculation (pi). The mice were euthanized on day 7 pi, their lungs were removed, and their lung viral titers quantified. Fifty female mice, of either strain 129/Sv (n = 15) or strain SJL/J (n = 35), were enrolled in the study of irradiation effects. The SJL/J mice were divided randomly into three groups of respectively 15, 10, and 10 mice. The 15 129/Sv mice and the group of 15 SJL/J mice were infected without undergoing any prior treatment. They were enrolled in the experiment to provide sets of resistant (SJL/J) and susceptible (129/Sv) non-irradiated mice with which to compare the principals (irradiated-infected mice). Both groups of 10 SJL/J mice were subjected to whole-body gamma irradiation (γ -WBI), followed four days later by intranasal instillation of either the virus-containing inoculum or a sterilized inoculum. The mock-infected mice were used to distinguish effects of irradiation from effects of infection. To irradiate the mice we followed exactly the same procedure as that validated previously for immunodepressing the SJL/J strain [41]. More exactly, we know the single γ-rays exposure at 9.02 Gy of SJL/J mice cause immediate, prolonged repression of leukopoiesis, as attested by a ∼99% reduction of the PBMC/circulating lymphocyte count. Eleven days post-irradiation a recovery trend is observed but recovery was only partial, since cell counts reach only ∼2% of their control value. Differential radiosensitivity also appears among cell types, NK cells being much more radioresistant than non-NK lymphocytes, and B lymphocytes more radiosensitive than T lymphocytes [41]. Live weight and plethysmographic values were recorded four successive times, once before and three times after virus inoculation (days 5, 6 and 7 pi). All mice were euthanized 7 days pi and their lungs sampled in order to assess virus dissemination (by immunofluorescence) and to measure the viral load.

### Mice, virus, and inoculation

The experiments were conducted with specific pathogen-free female inbred mice obtained from Charles River Laboratories. Cohorts of female F1 hybrids and F2 mice were produced in our own facilities, reciprocal crosses “129/Sv×SJL/J” and “SJL/J×129/Sv” (according to international nomenclature, strain which is the female parent is given first) being equally represented in the F1 cohort. The mice were made familiar with the experimental environment by placing them in the plethysmograph for 15 min a day, starting 3 days before inoculation. Housing, inoculation, data collection, and euthanasia procedures complied with National Institutes of Health guidelines, and the experimental protocol was approved by the Bioethics Committee of the University of Liege. PVM strain J3666 (generously supplied by A. Easton) was first passaged in 10-wk-old BALB/c mice and then grown once in BS-C-1 cells to produce the stock solution. The stock solution was then diluted to 10^−5^ in MEM, aliquoted, and stored at −80°C to serve as the inoculum. Randomly selected aliquots yielded highly reproducible titers on BS-C-1 cells, amounting to ∼2.10^4^ PFU/ml. The inoculation procedure consisted in slowly instilling 50 µl of viral suspension (∼10^3^ PFU) into the nostrils of the anesthetized mouse (10 mg.kg^−1^ xylazine and 50 mg.kg^−1^ ketamine, ip) maintained in a vertical position.

### Assessment of respiratory pattern and function

Respiratory pattern/function (RPF) values were measured with the two-chambered, whole-body plethysmograph devised by Buxco (model PLY-3351), using practical procedures, quality controls, and methods for raw data processing and respiratory flow curve analysis previously validated in the laboratory [42]. A series of parameters were measured directly on the basis of the thoracoabdominal flow curve: inspiratory time (TI), expiratory time (TE), tidal volume (TV), and time needed to exhale the first 30% of the TV (TE^30%^). Lastly, on the basis of the parameters measured above, the respiratory rate [RR = 60.000/(TI+TE)], the minute ventilation [MV = RR×TV], and the expiratory balance [EB = TE^30%^/TE] were calculated.

### Ethics statement

This study was carried out in strict accordance with the recommendations in the Guide for the Care and Use of Laboratory Animals of the National Institutes of Health and with the official Belgian guidelines (www.ejustice.just.fgov.be/mopdf/2010/05/14_1.pdf). The protocol was approved by the institutional Animal Care and Use Committees of University of Liege (Permit number: #499). As adoption of a biphasic expiratory pattern has been shown to announce death within ∼24 h, this qualitative sign was chosen, for humane reasons, as the endpoint of the experimental disease. Prior to each inoculation or euthanasia procedure, the animals were anesthetized by intraperitoneal injection of a mixture of ketamine (50 mg.kg^−1^) and xylazine (10 mg.kg^−1^).

### Assessment of lung lesions, virus dissemination, and virus yields

At selected time intervals (5, 6, and 7 days pi), mice were overdosed with sodium pentobarbital and exsanguinated by cutting the renal artery. The right lung was weighed, homogenized in PBS-BSA 1%, and clarified (3000 g for 10 min), and the supernatant was used for virus titration by plaque assay on BS-C-1 cells. Once quasi-confluent monolayers grown in 24-well plaques were obtained, the wells were filled with 200 µl lung homogenate (resulting from serial ten-fold dilutions). The viral suspensions were left to adsorb for 3 hours at 32°C and then the wells were washed with PBS and the cell monolayers covered with 1 ml of 0.6% agarose (w/v) in MEM containing 2% w/v FBS. After incubation at 32°C for 12 days, the agar overlay was removed and the remaining cells stained with crystal violet. Plaques were counted and the viral titer per gram right lung weight was calculated. The left lung was used for histopathology (hematoxylin and eosin staining) and the distribution of PVM antigens was examined by fluorescence microscopy, after sequential incubation of dewaxed sections with anti-PVM antiserum and FITC-conjugated antirabbit IgG antibody (Molecular Probes).

### Heritability and conformity with the additive-dominance model

Heritability in the broad sense was estimated from the increased variance found in the F2 generation compared to the F1. Individual scaling tests were used to test the conformity of the data with the additive-dominance model [43]. The test is based on the relationships between the generation means. The quantity C = 4
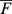
_2_−2
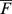
_1_−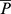
_1_−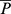
_2_ and its variance V(C) = 16 V(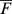
_2_)−4 V(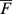
_1_)−V(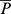
_1_)−V(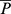
_2_) were calculated, and if the model is adequate, C equals zero within the limits of the sampling error. Afterwards, a joint scaling test was applied to the available generations to estimate the parameters *m* (a constant depending on the action of nonconsidered genes and nongenetic factors), *d* (the additive component), and *h* (the dominance deviation) and to compare the means observed for each generation with expected ones obtained from the three estimated parameters [43], [44], [45]. These three parameters were estimated by weighted least squares, and a χ^2^-test was used to test the goodness of fit of the additive-dominance model. The (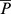
_2_-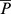
_1_)/Σd ratio (r_d_) measures the association of genes of like effects; r_d_ = 1 if all genes increasing/decreasing the trait are associated in one parental strain, and r_d_ = 0 if the contributing genes are distributed equally between the parental strains. The Σh/(r_d_ Σd) ratio (potence ratio) varies from 0 to ∞ (with the restriction that h≤|d| for each individual gene). If the *h* increments are balanced, i.e. if the sum of the dominance deviations which increase the traits equals the summed dominance deviations which decrease the traits, Σh = 0, no matter what the global dominance is. When the parental strains are not different because the genes involved are equally dispersed, r_d_ = 0 and the potence ratio = ∞. So, any observable potence ratio suggests that dominance of the involved genes acts in the same direction.

### Statistical analysis

A two-way analysis of variance was used to compare reciprocal F1 mice for all traits using a repeated daily measurements mixed linear model and a one way analysis was used to seek a generation effect on log_10_ means of lung virus titers. The lung virus loads measured in the immunosuppression study were analyzed by two-way analysis of variance to examine the effects of the time elapsed from irradiation/inoculation and of mouse pretreatment (irradiation or not) [46]. A two-way analysis of variance for repeated measurements was used to examine the effects of the time elapsed from inoculation of (i) generation (parental, F1, F2) or pretreatment, and of (ii) the interaction between generation or pretreatment and time on body weight and RPF values [47]. Pairwise comparisons of least square means of fixed effects were made with Student's t-test. The daily evolution of body weight and RPF traits were compared among the four generations by (i) the least squares means (lsmeans) for generations, (ii) the significance of the differences between the two parental strains and offspring, (iii) the significance of the linear interaction between generations and days, and (iv) the regression slopes (b) on days post-inoculation. Comparisons yielding P values<0.05 were considered statistically significant.

## Results

### Descriptive analysis of PVM-susceptibility

#### Morbidity

**Figure 1:**
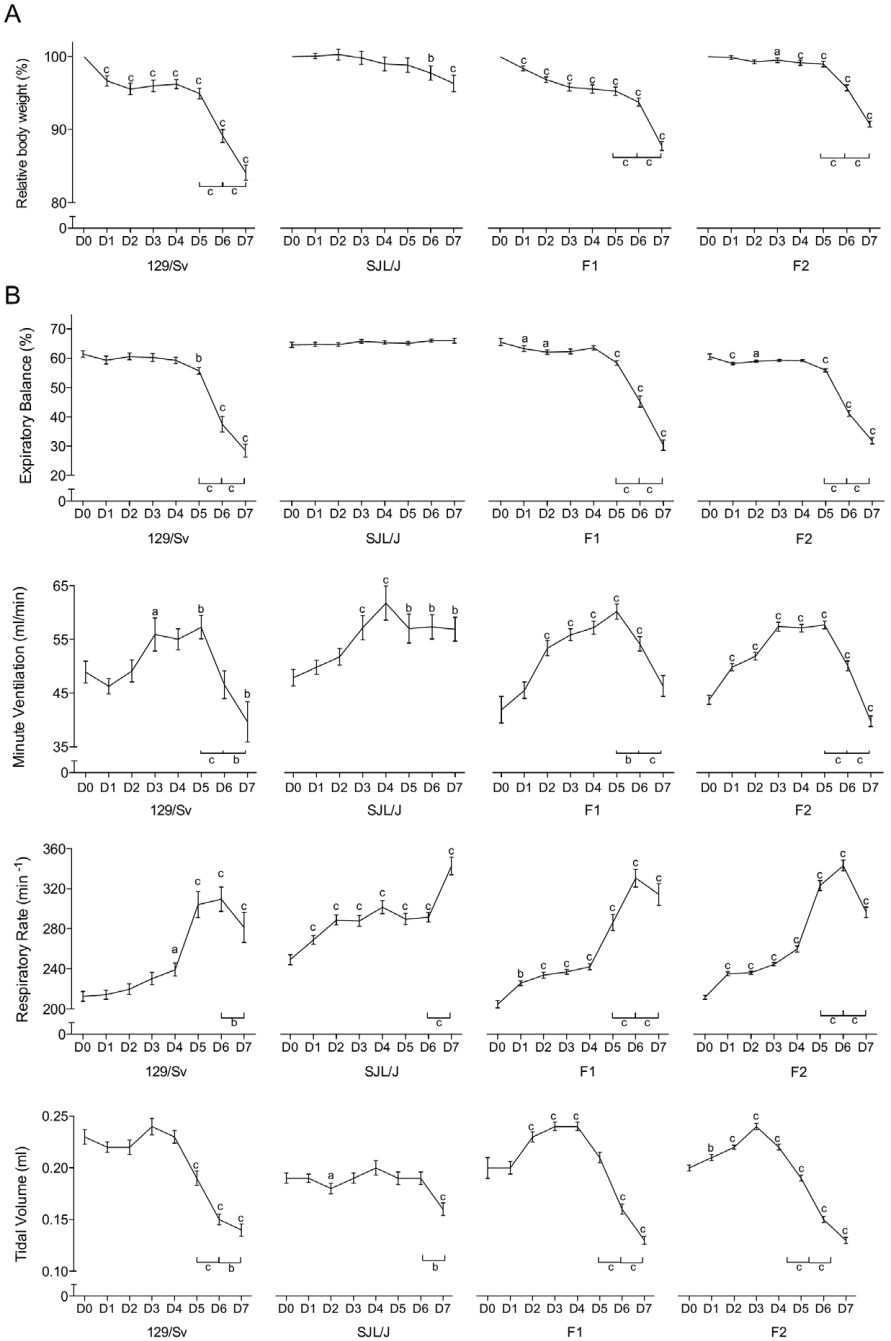
Morbidity and respiratory pattern values. (A) Evolution of daily body weight within each generation from PVM inoculation to 7 days later (% of the pre-inoculation value, mean ± SEM). Means significantly different from the corresponding pre-inoculation values (on day 0) are indicated with a (p<0.05), b (p<0.01), or c (p<0.001). Significant day-to-day changes after day 5 pi are indicated also (bottom-right). (B) Respiratory dysfunctions were measured using a double chamber plethysmograph. Before and at selected time points after intranasal inoculation of PVM, minute ventilation (MV), expiratory balance (EB), respiratory rate (RR) and tidal volume (TV) were determined within each generation (mean ± SEM). Means significantly different from the corresponding pre-inoculation values (on day 0) are indicated with a (p<0.05), b (p<0.01), or c (p<0.001). Significant day-to-day changes after day 5 pi are indicated also (bottom-right).

The evolution of body weight (BW) after inoculation significantly differed among generations (Table 1, interaction for BW: p<0.001). Susceptible and resistant control groups matched that reported previously [40]: 129/Sv lost weight dramatically throughout the observation period (relative BW lsmean = 94%, b = −1.7% per day), with a nadir on day 7 pi (−16%), whereas SJL/J BW practically remained stable (relative BW lsmean = 99%, b = −0.5% per day) (Table 2, Fig. 1A). Both the F1 (relative BW lsmean = 95.4%, b = −1.4% per day) and the F2 (relative BW lsmean = 97.8%, b = −0.6% per day) generations showed an intermediate pattern, with respective losses of ∼12 and ∼9% on day 7 pi (Tables 1 and 2, Fig. 1A).

**Table 1:** Differences between least squares means for generations over the entire post-inoculation period, and significance of the interaction between generations and days post-inoculation.

Traits	129 vs. SJL	129 vs. F1	129 vs. F2	SJL vs. F1	SJL vs. F2
BW (%)	4.9***	−1.3*	−3.8***	3.6***	1.1 ^NS^
Interaction for BW	***	***	***	***	***
MV (ml/min)	5.1**	−2.0^NS^	−1.1 ^NS^	3.1*	4.0**
Interaction for MV	***	*	NS	***	***
EB (%)	12***	−4**	−1^ns^	9***	12***
Interaction for EB	***	NS	NS	***	***
RR (min^−1^)	38.0***	−7.4 ^NS^	−16.9**	30.6***	21.1***
Interaction for RR	***	*	NS	***	***
TV (ml)	−0.014**	−0.001 ^NS^	0.006 ^NS^	−0.015**	−0.008*
Interaction for TV	***	*	NS	***	***
LVL^d7^ (log_10_ PFU/g tissue)	3.84***	−1.24***	−1.60***	2.60***	2.23***

The evolution (profiles) during the 7 days post-inoculation of the 129/Sv and the SJL/J parental strains were compared to the evolution (profiles) of the F1 and the F2 generations. A non significant generation-day interaction means that the profiles of the two generations involved are parallel whereas a significant one means a different pattern.

BW, relative body weight; LVL^d7^, lung viral load at day 7 pi; MV, minute volume; EB, expiratory balance; RR, respiratory rate; TV, tidal volume.

*** , p<0.001;

** , p<0.01 and

* p<0.05. NS, non significant.

**Table 2:** Least squares mean of traits for generations over the entire post-inoculation period with its standard error (lsmean, SE), traits linear regression slopes (b) on the days post-inoculation with its level of significance, and minimum and maximum daily standard deviation (SD, min-max).

Traits		129/Sv	SJL/J	F1	F2
BW (%)	lsmean, SE	94 (0.005)	99 (0.007)	95.4 (0.004)	97.8 (0.003)
BW	b	−1.7***	−0.5**	−1.4***	−0.6***
BW	SD, min-max	3.19–4.69	1.86–5.81	2.17–4.08	3.83–6.6
EB (%)	lsmean, SE	53 (0.008)	65 (0.004)	56 (0.005)	53 (0.003)
EB	b	−3.8***	0.2 ^NS^	−4.0***	−3.5***
EB	SD, min-max	5–13	2.6–4.4	4.8–12.3	4.6–15.8
MV (ml/min)	lsmean, SE	49.9 (1.33)	55.0 (1.32)	51.8 (0.64)	51.0 (0.42)
MV	b	−0.22^NS^	1.40***	1.17***	−0.08^NS^
MV	SD, min-max	7.48–16.32	6.68–16.15	7.15–16.05	10.29–15.02
RR (min^−1^)	lsmean, SE	252 (5.0)	290 (4.1)	259 (2.6)	269 (1.8)
RR	b	15.0***	9.3***	17.4***	17.1***
RR	SD, min-max	24.53–68.29	21.97–44.08	14.53–64.75	23.86–77.32
TV (ml)	lsmean, SE	0.202 (0.003)	0.188 (0.003)	0.202 (0.002)	0.195 (0.002)
TV	b	−0.012***	−0.0015^NS^	−0.009***	−0.011***
TV	SD, min-max	0. 026–0.04	0.021–0.036	0.025–0.063	0.039–0.049

BW, relative body weight; EB, expiratory balance; MV, minute volume; RR, respiratory rate; TV, tidal volume.

*** , p<0.001;

** , p<0.01. NS, non significant.

#### RPF values

The functional consequence on the respiratory function of the virus induced lung lesions was investigated and several respiratory parameters were recorded. Again the parental lines showed the expected disease-associated alterations [40], with a dramatically lengthened expiratory emptying for 129/Sv: on day 7 pi, EB = ∼50% of baseline value (p<0.001), contrasting with a stable expiratory compartmentation in SJL/J (Table 2, b = 0.2; Fig. 1B). The 129/Sv-like expiratory pattern was also clearly visible in both F1 and F2 generations, with a dramatic lengthening of the time required to exhale the last part of the TV: on day 7 pi, EB = ∼47 and 53% of baseline value, respectively (p<0.001) (Table 1, Fig. 1B). The ventilatory patterns of both F1 and F2 generations rather paralleled that displayed by 129/Sv, with an initial gradual increase in MV up to day 5 pi, followed by a steep decay on and after day 6 pi (Tables 1 and 2, Fig. 1B). As in the 129/Sv parental line, this was accomplished via a bell-shaped evolution of RR combined with a continuous decrease in TV (Tables 1 and 2, Fig. 1B). With respect to pre-inoculation values, the parental lines displayed significantly different RPF values (p<0.001), while achieving a similar MV. Most baseline values of both crosses were not different from those of 129/Sv (p>0.05).

#### Lung viral load

**Figure 2:**
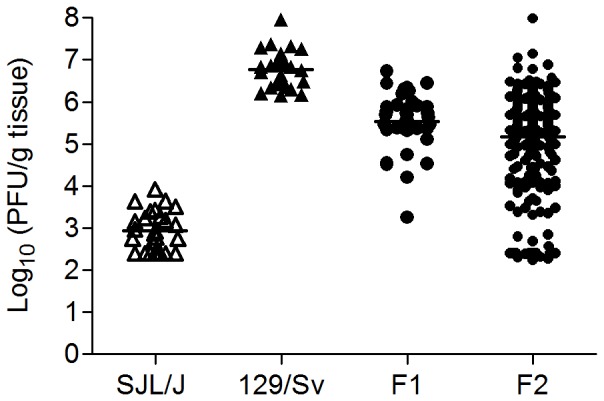
Lung viral load 7 days after PVM inoculation (LVL^d7^, log_10_ PFU/g). Each symbol corresponds to a single mouse. Generation-specific means are indicated (horizontal lines). Each mean was significantly different from the other three (p<0.001). Means ± SEM: 2.94±0.09 (SJL/J), 6.77±0.09 (129/Sv), 5.53±0.1 (F1) and 5.17±0.07 (F2).

To maximize scattering of the quantitative trait to be recovered, lung viral titers were measured 7 days pi, i.e. at time point where the greatest difference was detected between parental strains [40]. Lung virus titers in both crosses were significantly lower (p<0.001) than the titer measured in 129/Sv and significantly higher (p<0.001) than that typical of SJL/J (Fig. 2).

### Genetic analysis of PVM-susceptibility

**Figure 3:**
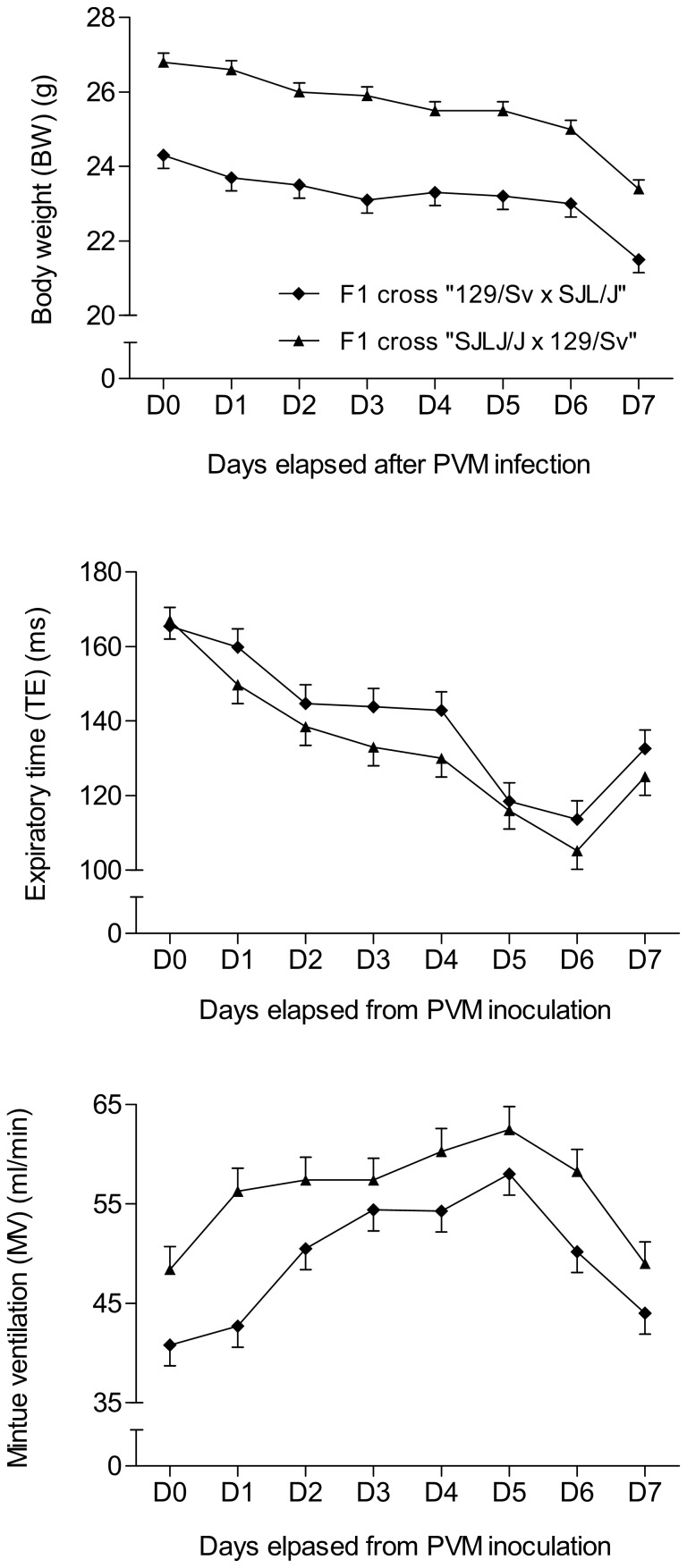
Significant differences between the two reciprocal F1 crosses (“129/Sv×SJL/J” vs. “SJL/J×129/Sv”). Significant differences appeared over the whole period of observation post-inoculation for (a) the body weight (BW) (p<0.001), (b) the expiratory time (TE) (p<0.05) and (c) the minute ventilation (MV) (p<0.001). Means ± SEM are represented.

**Figure 4:**
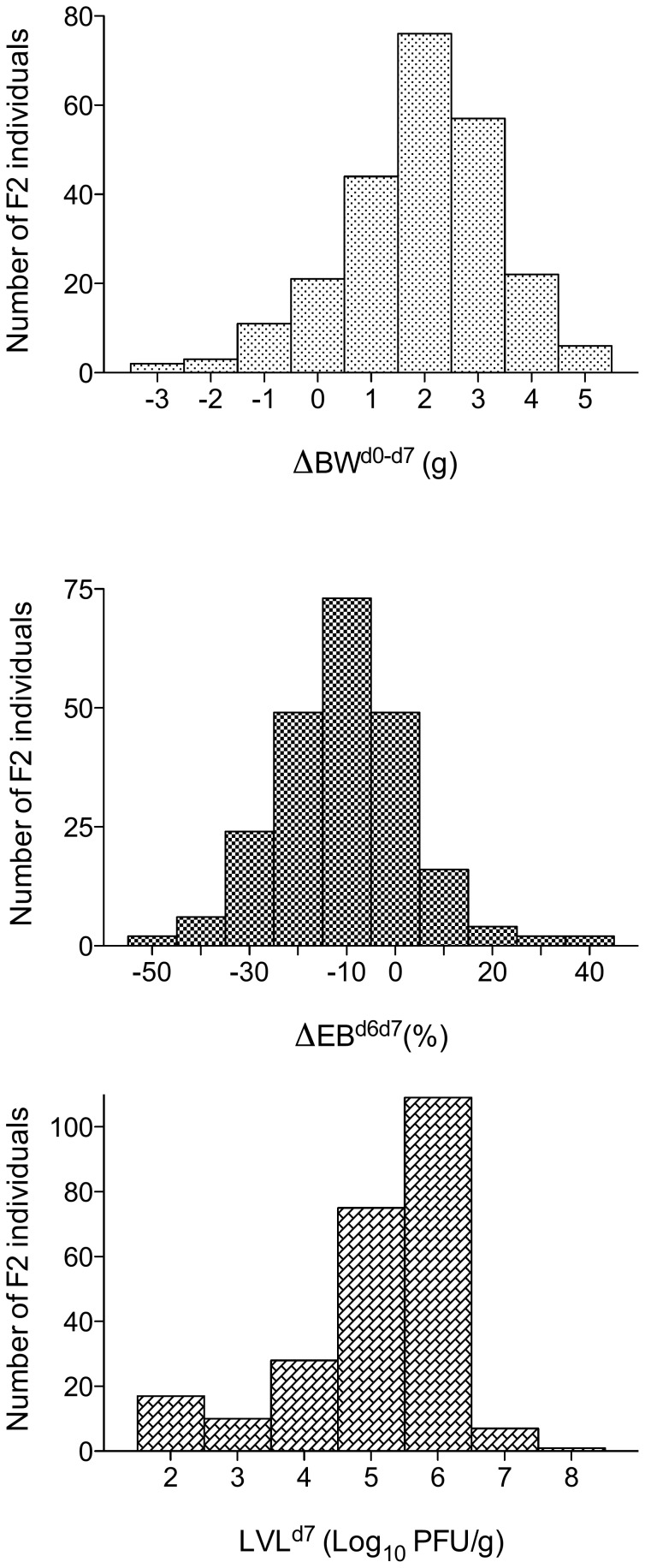
Distribution in the F2 generation of the three most pertinent PVM-susceptibility traits: (a) ΔBW^d0–d7^, the body weight loss at day 7 pi (g), (b) ΔEB^d6–d7^, the expiratory balance loss from day 6 to day 7 (%) and (c) LVL^d7^, the lung viral load on day 7 (log_10_ PFU/g).

Over the entire period of post-inoculation observation, significant differences only appeared between the two reciprocal parental crosses (“129/Sv×SJL/J” and “SJL/J×129/Sv”) for BW (p<0.001), TE (p<0.05), and MV (p<0.001) (Fig. 3). However, interaction between reciprocal crosses and days post-inoculation was not significant. Thus, data for BW, TE and MV were pooled when analysing the evolution with time post-inoculation in F1 generation (Fig. 1). The three most pertinent traits in terms of PVM-susceptibility were chosen for further analysis: day 7 pi BW loss (for global morbidity, ΔBW^d0–d7^), EB loss from day 6 to day 7 pi (for global respiratory failure, ΔEB^d6–d7^), and lung viral load (for global lung permissivity towards PVM replication, LVL^d7^). F1 mean values were intermediate between the two parental lines for ΔBW^d0–d7^ and LVL^d7^ (Figs. 1A and 2, Table 3). In the F2 generation, the standard deviations of the three variables were greater than in either parental strains or F1 (Table 3, Fig. 2) and their distributions were continuous and unimodal (Fig. 4). The broad-sense heritability estimate was high for LVL^d7^ (h^2^ = 0.66) and ΔBW^d0–d7^ (h^2^ = 0.38) and moderate for ΔEB^d6–d7^ (h^2^ = 0.23).

**Table 3:** Observed means with standard deviations, and predicted means of most pertinent PVM-susceptibility traits.

Generation	LVL^d7^ (log_10_ PFU/g)		ΔBW^d0–d7^ (%)		ΔEB^d6–d7^ (%)	
	Body weight loss					
	Obs ± SD	Predicted	Obs. ± SD	Predicted	Obs. ± SD	Predicted
	± SD					
P1 (SJL/J)	2.9±0.47	2.9	3.9±5.8	1.9	0.3±4.0	0.2
P2 (129/Sv) ((((129/Sv)	6.8±0.44	6.8	16.1±4.7	14.1	11.1±9.7	10.7
F1	5.5±0.66	5.5	12.2±4.1	10.5	14.8±12.3	14.3
F2	5.2±1.13	5.2	9.25±6.6	10.5	9.7±14.0	9.9

LVL^d7^, day 7 pi lung viral load (log_10_ PFU/g); ΔBW^d0–d7^, body weight loss as measured 7 days pi (%); ΔEB^d6–d7^, change in expiratory balance between days 6 and 7 pi (%).

P1, PVM-resistant parental strain (SJL/J) and P2, PVM-susceptible parental strain (129/Sv). Predicted means were calculated by the joint scaling test. Obs., observed.

For such quantitative traits, the effect of the genes involved can be described by the parameters Σd and Σh, where *d* represents the fixable additive component of the mean and *h*, which depicts the dominance features of the genes, represents the unfixable heritable component of the mean. The values of these parameters were estimated and used to calculate the predicted mean of each trait for each generation (Table 4). For ΔEB^d6–d7^ and LVL^d7^, the C values resulting from individual scaling tests were not significantly different from zero (p>0.05), suggesting that the mode of inheritance of these two PVM-susceptibility indices fits the additive-dominance model (Table 4). This was confirmed further by the joint scaling test (Table 4). In contrast, both the individual and the joint scaling test unambiguously refuted this pattern for ΔBW^d0–d7^, suggesting the presence of non-allelic interactions that we were unable to confirm, because backcross generations were not available. Values of Σd and Σh were approximately equal, with a positive Σh for all three traits. A dominance pattern was thus present, and the alleles increasing PVM-susceptibility appeared dominant. The r_d_ ratio equaled ∼1 for all three traits (Table 5); this means that among the genes considered, the alleles for susceptibility and for resistance to PVM are associated, respectively, in the 129/Sv and SJL/J strains. The potence ratio for the three traits ranged from 0.35 to 1.65, suggesting that dominance acts in the same direction at most of the loci. In summary, PVM-susceptibility is a polygenic trait, the susceptibility alleles are dominant, and most of the alleles operating in the same direction are associated.

**Table 4:** PVM-susceptibility values across generations fit with additive-dominance model.

Traits	Individual scaling test		Joint scaling test				
	C ± s.e.	t test	*m* ± s.e.	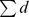 ± s.e.	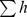 ± s.e.	χ^2^_(1)_	P
**LVL^d7^**	−0.09±0.38	N S	4.85±0.06	1.92±0.06	0.67±0.11	0.06	0.8
**ΔBW^d0–d7^**	2.81±0.61	P<0.05	1.75±0.14	1.37±0.17	0.94±0.24	21.5	<0.001
**ΔEB^d6–d7^**	−0.02±0.061	N S	0.05±0.01	0.05±0.01	0.09±0.02	0.11	0.74

LVL^d7^, day 7 pi lung viral load; ΔBW^d0–d7^, body weight loss as measured 7 days pi; ΔEB^d6–d7^, change in expiratory balance between days 6 and 7 pi. Scaling tests according to Mather and Jinks, 1982. See Materials & Methods section for key.

**Table 5:** PVM-susceptibility gene distribution between parental strains.

Traits	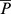 _1_ (SJL/J)		( 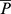 _2_− 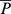 _1_)	r_d_	Potence
LVL^d7^	2.94	6.77	3.84	0.99	0.35
ΔBW^d0–d7^	0.76	3.56	2.80	1.02	0.69
ΔEB^d6–d7^	0.003	0.11	0.11	1.04	1.65

LVL^d7^, day 7 pi lung viral load; ΔBW^d0–d7^, body weight loss as measured 7 days pi; ΔEB^d6–d7^, change in expiratory balance between days 6 and 7 pi. P1 and P2, mean value of PVM-resistant (SJL/J) and PVM-susceptible (129/Sv) strain, respectively. Testing according to Mather and Jinks (1982). See Materials & Methods section for key.

### Effect of WBI on PVM-susceptibility in the resistant line

**Figure 5:**
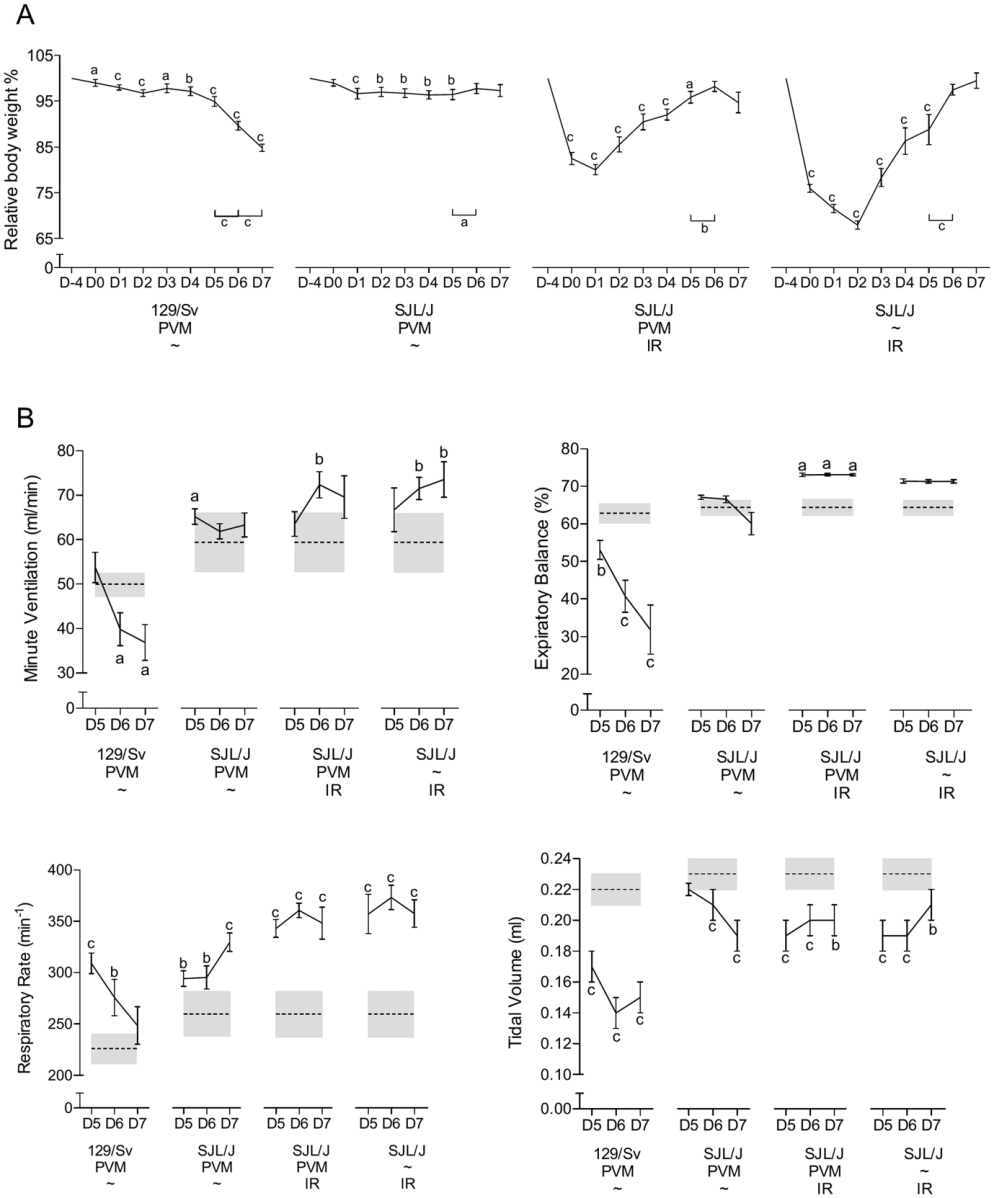
Effect of whole-body γ-irradiation. (A) Evolution of body weight from PVM or mock (∼) inoculation to 7 days later in non-irradiated (∼) 129/Sv (susceptible) and SJL/J (resistant) controls and in irradiated SJL/J principals (mean ± SEM). Means significantly different from the corresponding pre-inoculation values (on day −4) are indicated with a (p<0.05), b (p<0.01), or c (p<0.001). Significant day-to-day changes after day 5 pi are also indicated (bottom-right). IR, whole-body γ-irradiation. (B) Respiratory dysfunctions were measured using a double chamber plethysmograph. Before and at selected time points after intranasal inoculation of PVM or sterilized inoculum (∼), minute ventilation (MV), expiratory balance (EB), respiratory rate (RR) and tidal volume (TV) values were determined for each group: the non irradiated infected 129/Sv (susceptible) and SJL/J (resistant) controls, the irradiated infected SJL/J principals and the irradiated mock infected (∼) SJL/J (mean ± SEM). Means significantly different from the corresponding pre-inoculation values (on day −4) are indicated with a (p<0.05), b (p<0.01), or c (p<0.001). Gray boxes show the means (dotted line) and SEM (height) of the corresponding pre-inoculation values. IR, whole-body γ-irradiation.

Mortality/morbidity — All the SJL/J mice (PVM-resistant) survived PVM infection, including those irradiated before inoculation, whereas the cumulated mortality rate was 30% among PVM-infected non-irradiated 129/Sv mice. Pretreatment (none vs. γ-WBI), the nature of the inoculum (mock vs. PVM), and their interaction had a significant effect on the BW course over time (p<0.001, Fig. 5A). Among the non-irradiated controls, PVM-infected SJL/J mice practically maintained their BW throughout the observation period, whereas a continuous decrease was recorded for 129/Sv, slow till day 5 pi and much faster thereafter, with a nadir on the last study day (BW loss ∼15%). In the principals (irradiated SJL/J), γ-WBI significantly altered the BW course, but with a different kinetics: these mice showed first a severe loss starting the day after irradiation (minus ∼17 and ∼26%), and then a steep recovery from day 1–2 pi to day 6 pi.

#### RPF values

As between-strain differences in RPF values reached a maximum between days 5 and 7 pi [40], data collection was restricted to four time points: day 4 before inoculation and days 5, 6, and 7 pi. Regarding the ventilatory pattern, three specific types of response were observed (Fig. 5B). PVM-susceptible 129/Sv mice showed a stable MV up to day 5 and then a steep decrease, with a nadir on day 7 pi (p<0.05). The initial stable MV was achieved by a stable RR-TV combination, whereas the hypoventilation period was characterized by a dramatically collapsed TV (p<0.001) not sufficiently balanced by an RR increase (Fig. 5B). The PVM-resistant non-irradiated SJL/J cohort showed a constant MV throughout the observation period, achieved by a well-matched RR-TV combination (Fig. 5B) the more the TV decreased, the more the RR increased. Irradiated PVM-resistant SJL/J mice, infected or not, showed identical ventilatory patterns: a stable MV until day 5 pi and then an increase (Fig. 5B), the latter being achieved by an increase of both TV and RR (Fig. 5B). The expiratory limb of the airflow curve displayed two distinct shapes with a clear difference between 129/Sv (dying) and SJL/J (surviving) mice. In 129/Sv, a dramatic abatement of the EB index was seen from day 5 pi onward, whereas in SJL/J mice it remained unchanged or even increased (Fig. 5B). The EB abatement reveals a recompartmentation of expiration in the 129/Sv mice, which took longer and longer to exhale the last part of the TV because of premature lower airway closure.

#### Histological alterations

**Figure 6:**
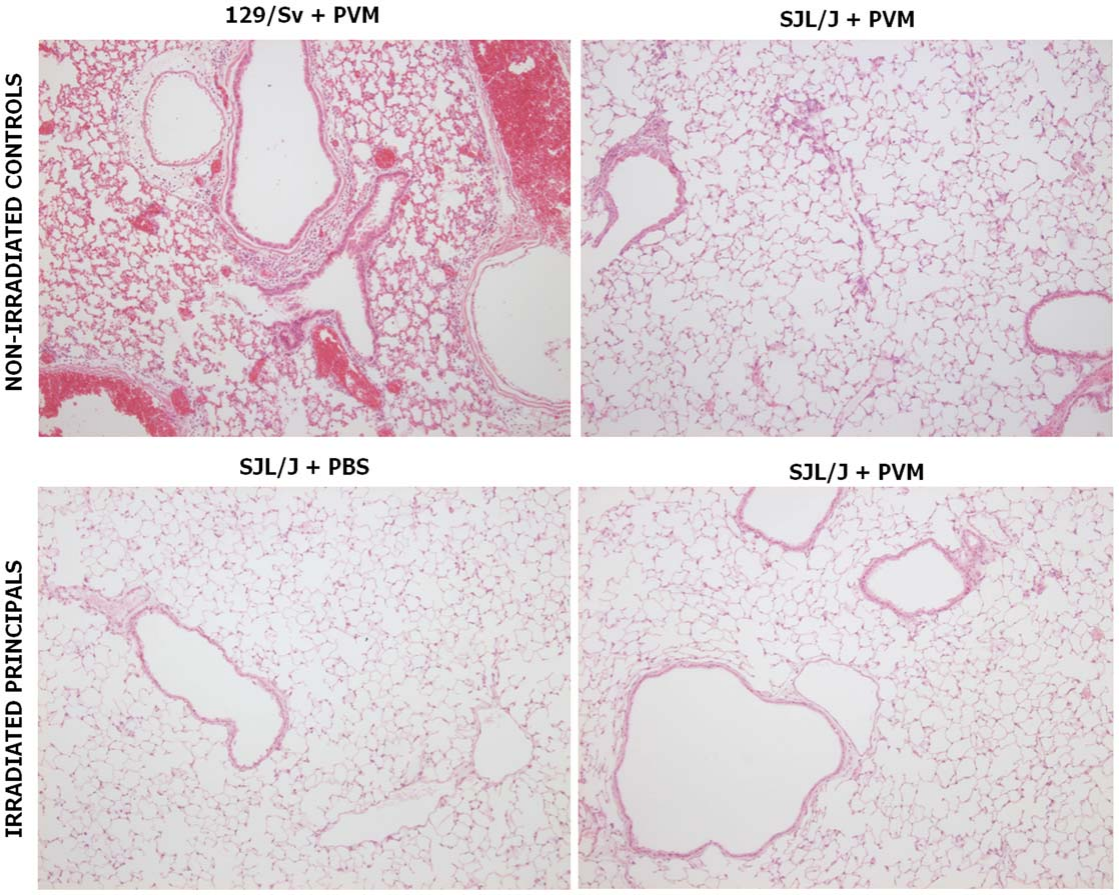
Typical histological views of mouse lungs 6 days after PVM or mock inoculation. Congestion and inflammatory infiltrates are prominent in non-irradiated infected 129/Sv, discrete in non-irradiated infected SJL/J, and absent in irradiated infected SJL/J. See text for details. Lung sections were stained with hematoxylin and eosin for histological evaluation, magnification ×100.

Three distinct histological pictures were readily distinguished by unaware examiners. Those typical of non-irradiated, infected 129/Sv and SJL/J were in total agreement with published data [40]: SJL/J lungs showed slight multifocal mononuclear pneumonitis, with scarce, incomplete peribronchic/vascular cuffs, whereas the lungs of 129/Sv mice showed congestion, diffuse mononuclear interstitial pneumonitis, and multifocal mononuclear alveolitis, with severe cuffing of the bronchi and vessels (Fig. 6). In contrast, inflammatory cell infiltrations were absent from the lungs of irradiated SJL/J, infected or not, except for a few isolated alveolar macrophages (Fig. 6). These latter histological pictures, never reported previously, are compatible with γ-WBI-associated panleukopenia.

#### Virological data

**Figure 7:**
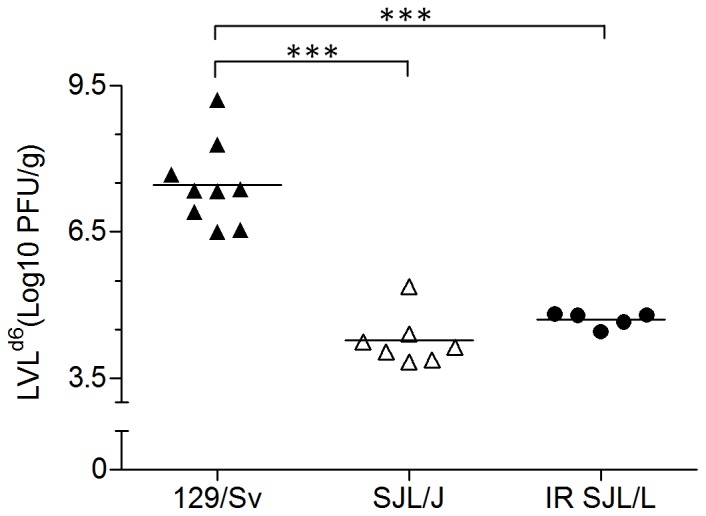
Immunosuppression by whole-body γ-irradiation does not exacerbate PVM replication. Each symbol corresponds to the lung viral load 6 days after inoculation (LVL^d6^) of a single mouse and specific means of the different groups (susceptible 129/Sv, resistant SJL/J and irradiated (IR) resistant SJL/J) are indicated (horizontal lines). ***, p<0.001.

**Figure 8:**
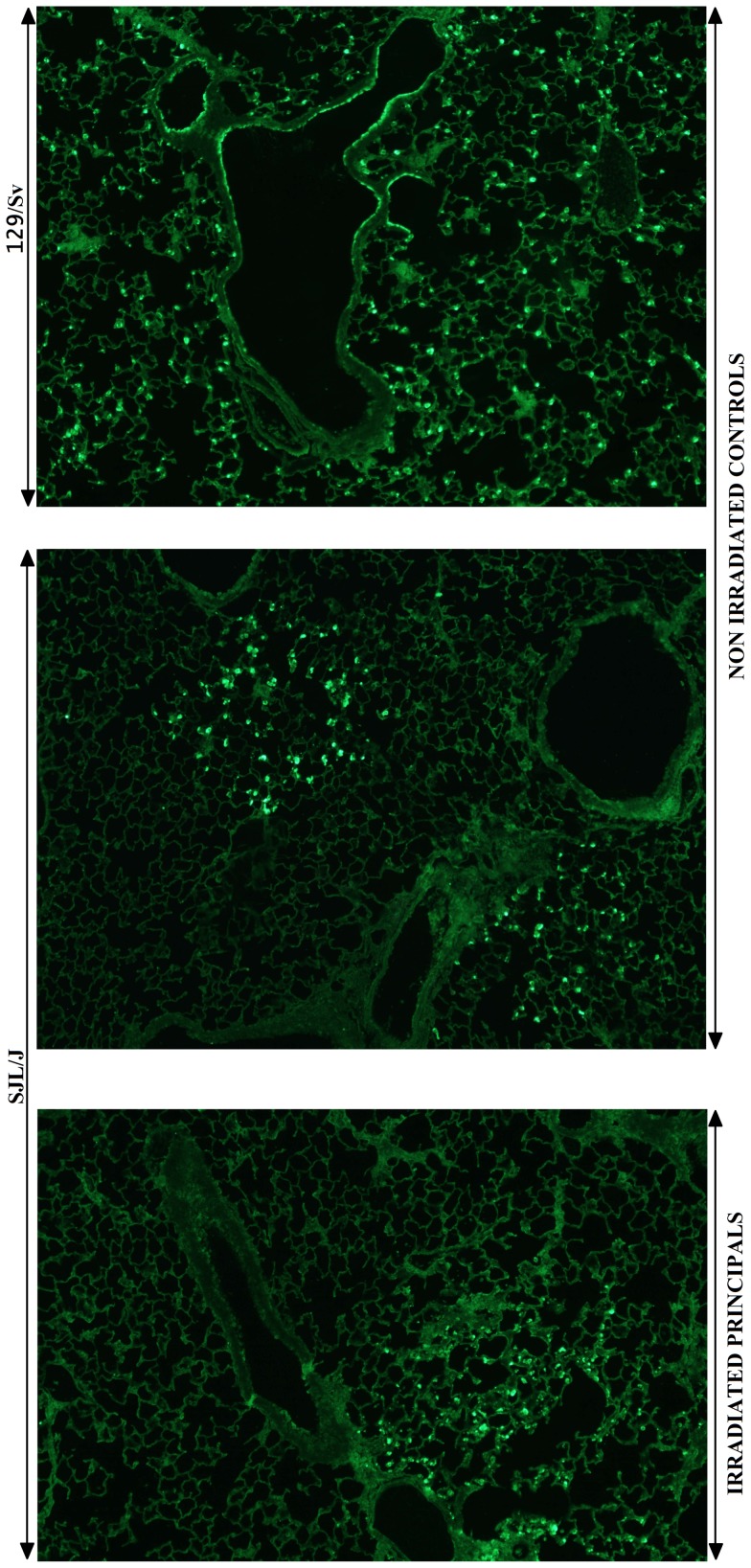
Immunosuppression by whole-body γ-irradiation does not result in increased dissemination of PVM throughout the lungs. Viral antigens were detected by indirect immunofluorescence, after sequential incubation of the dewaxed lung sections with anti-PVM antiserum and FITC-conjugated antirabbit IgG antibody (Molecular Probes). Staining was absent when anti-PVM rabbit polyclonal primary antiserum was replaced with a nonpertinent rabbit serum.

As peak lung viral loads occurred 6 days pi in both strains [40], infectious virus particle titration and antigen distribution were restricted to this specific time point (Figs. 7–8). As expected, 129/Sv mice displayed the highest viral loads and SJL/J, the lowest. Absolute values agreed with previously reported reference titers [40]. The lung viral load recovered from irradiated SJL/J mice was dramatically lower than that recovered from 129/Sv mice (p<0.001) and matched perfectly that found in non-irradiated SJL/J mice (Fig. 7). The topological distribution of viral antigens within the lungs, as visualized by immunofluorescence, also appeared very different according to the strain (Fig. 8), but irradiated and non-irradiated PVM-resistant SJL/J mice showed the same distribution. From day 6 pi onward, the 129/Sv strain typically displayed virus-positive bronchial/bronchiolar epithelial cells, type 1 and 2 pneumocytes, and macrophages homogeneously distributed throughout the lungs. By comparison, only a few and far-between virus-positive foci were visible in SJL/J lungs, consisting of infected type 1 and 2 pneumocytes and some alveolar macrophages. No virus-positive cells were detected in mock-infected mice.

## Discussion

We have shown previously that in mice, the genetic background influences significantly their resistance/susceptibility to bronchiolitis and pneumonia caused by PVM. Focusing on a panel of six inbred lines [40], strain SJL/J emerged as the most resistant and strains DBA/2 and 129/Sv as the most susceptible, the other four strains (BALB/c, FVB/J, C3H/HeN, C57BL/6) showing various intermediate levels of resistance. Such a continuous distribution of phenotypes is suggestive of a polygenic genetic determinism of the underlying antiviral mechanism(s). Here we have examined the segregation of phenotypes in the descendants of the two extreme strains and we have also immunodepressed strain SJL/J so as to better characterize its resistance to PVM.

### The resistance of SJL/J is polygenic and recessive

Transmission of resistance/susceptibility to viral infections [48] might be either monogenic, as in the case of resistance to influenza A viruses in mouse [49], or polygenic [50], [51]. Three traits representative of resistance/susceptibility to PVM were used in our genetic analysis: LVL^d7^ (lung permissivity to PVM replication), ΔEB^d6–d7^ (respiratory failure), and ΔBW^d0–d7^ (morbidity). F1 mean values were intermediate between the two parental ones (Table 3). In the F2 generation, for these three variables as for the other RPF variables (Table 2), the variability was greater due to the segregation of genotypes compared to the parental strains and F1, in which only variance of environmental origin is present (Table 3, Fig. 2). F2 distributions were continuous and unimodal (Fig. 4). All these features are typical of a complex, polygenic determinism [52], [53], as confirmed by the heritability values. Indeed, if the inheritance was monogenic, the F2 distributions should be a mixture of non overlapping P1, F1 and P2 populations in mendelian proportions 1∶2∶1 in case of partial dominance and, at least bimodal with two populations in proportions 3∶1, as far as there is a complete dominance. Dissection of the concerned genetic component reveals a dominance effect, an additive effect, and in the case of ΔBW^d0–d7^, a possible non-allelic interaction. More precisely, the sum of the individual genes dominance deviations in the heterozygous F1 (Σh), deviates for the three traits in the direction of increasing lung viral load on day 7 pi (LVL^d7^), BW loss at day 7 pi (ΔBW^d0–d7^) and EB loss from day 6 to day 7 (ΔEB^d6–d7^), that is in the direction of susceptibility which is thus dominant; resistance being recessive. In other words, the F1 resembles more the susceptible 129/Sv strain (Figs. 1 and 2). Furthermore, the dominance/recessivity effects of all the genes concerned operate in the same direction. In other words, at the genes underlying the resistance/sensitivity phenotype, the resistance alleles are associated in SJL/J whereas the sensitivity alleles are associated in 129/Sv. Also noteworthy is the difference between reciprocal crosses in the F1 (“129/Sv×SJL/J” and “SJL/J×129/Sv”) established for BW, TE, and MV. This suggests that these traits are subject to a maternal effect or to parental imprinting [54]. As the alleles of inbred murine lines are fixed, pseudo-imprinting can be ruled out [55]. In summary, PVM-susceptibility is a polygenic trait, the susceptibility alleles are dominant, and most of the alleles operating in the same direction are associated. As attested by the continuum of the resistance/susceptibility phenotypes shown among six inbred lines [40], the polygenic feature (also found for example in the case of the infection by the mouse adenovirus-1 [51] or by the Theiler's virus [50]) can be generalized while the recessivity of the resistance could not be applied to crosses implicating different inbred strains. Indeed, strain specific combinations of alleles could generate or change (intra- and interloci) interactions and/or induce variation in additive effects, modifying the genetic mode of transmission of the resistance genes in the F1. To our knowledge, there is no publication available in which susceptibility to PVM is compared between laboratory and wild mice. Antibodies to PVM were detected in the blood of wild animals from subfamilies *Murinae* and *Arvicolinae*, along with a negative relationship between seroprevalence and population size [56]. There is thus no doubt that these natural populations are susceptible to PVM, but no solid information is available on the relative severity of the disease in such or such subpopulation. As the resistance/susceptibility phenotype is a polygenic trait, one can suggest that genotypes with combinations of alleles resulting in very susceptible phenotypes are probably eliminated in nature. On the other hand, there are probably many allele combinations that are compatible with survival in nature, explaining why “PVM-susceptibility” alleles at some loci could have persisted in wild populations. Furthermore, some PVM-susceptibility alleles may function as resistance alleles for other pathogens or in other situations that would generate a selective advantage to heterozygotes. Coming back to SJL/J, the accumulation of several recessive alleles in this line is the consequence of a totally artificial selection and, for the reasons stated above, it is not sure that this genotype would be advantageous in nature. Lastly, the fact that the parental strains showed significant differences in some basal plethysmographic values (measured before infection) suggests that a fraction of the genetic component underlying resistance/sensitivity might concern the intrinsic structural or functional characteristics of the respiratory system.

### Resistance of SJL/J is not reduced by γ-WBI

In most cases reported in the literature, virus-infected mice having undergone an immunosuppressive procedure or having received immunosuppressor treatment show amplified virus replication, exacerbated lung dysfunction, more serious symptoms, and increased mortality [57], [58], [59]. The present data collected on SJL/J are not compatible with this scenario. Like their non-irradiated counterparts, all of the irradiated mice survived the infection, whereas the non-irradiated sensitive (129/Sv) mice died by the end of the test period. The irradiation procedure did cause a body weight loss, but the mice recovered their normal weight in the course of the infection, while the live weight of the non-irradiated sensitive mice continued to drop drastically. Functionally (RPF) and histopathologically, infected SJL/J mice showed the same profile whether they had been irradiated or not, whereas the infected 129/Sv mice reproduced exactly the expected major alterations [40]. Nor did γ-WBI affect the distribution of viral antigens in the lungs, which remained focal and discrete in contrast to the presence of virus observed throughout the lungs in 129/Sv. Lastly, despite a moderate rebound observed at the end of the experiment, the level of viral amplification remained persistently low in the lungs of irradiated SJL/J mice. The resistance of the irradiated mice could not be due to failure of the inoculation, since (i) viral amplification was observed in their lungs 7 days post-inoculation and (ii) functional and morphological alterations typical of the disease were identified in both sensitive (129/Sv) and resistant (SJL/J) non-irradiated controls. Consequently, our results suggest that mechanisms involving radiosensitive cells play no role in resistance. It is true that irradiation alone does alter the respiratory function (RPF), but this was expected [60], [61] and it is not believed to reflect the presence of epithelial lesions. Besides, the bronchiolar and pulmonary epithelia of our irradiated mice showed no histological alterations (Fig. 6) and remained perfectly capable of amplifying the virus, as attested by the moderate replicative rebound measured in irradiated mice at the end of the experiment. The γ -WBI procedure applied here was initially devised expressly for immunodepressing SJL/J mice [41]. It causes circulating levels of T and B lymphocytes and NK cells to collapse, with nadirs on day 4 post-irradiation [41] (i.e. the very day that PVM was inoculated). These results suggest that these cell actors are not involved in the resistance of strain SJL/J to PVM. Interestingly, neutrophils, which are among the cells early recruited after PVM infection [38], were also totally absent from the lung parenchyma of irradiated and infected SJL/J mice (Fig. 6). Despite the lack of direct counts, this finding is consistent with other reports [62], [63], [64]. Our immunofluorescence observations show that SJL/J can circumscribe the foci of viral replication, in contrast to 129/Sv (Fig. 8), BALB/c, C3H/HeN, DBA/2, and C57BL/6 [40], and FVB/J (Dermine, personal communication). Taken together, our data thus suggest that in SJL/J, radioresistant cells are responsible for developing an antiviral mechanism capable of stopping the centrifugal dissemination of virus from the primordial replication foci. This mechanism is not adaptive, since it is insensitive to whole-body irradiation. Our observations and our interpretation thereof are consistent with previous findings casting doubt on the hypothesis of T-lymphocyte-dependent resistance: (i) that in SJL/J, the repertoire of TCR V_β_ genes shows a very large deletion [65] and (ii) that PVM-specific CD4+ et CD8+ T lymphocytes are inactivated by PVM itself [66], [67]. Our data on resistance-trait segregation in hybrids further support this view. None of the resistance/susceptibility traits measured is totally transmitted to the F1 generation (Figs. 1 and 2), the intermediate phenotypes observed in F1 are therefore incompatible with an involvement of the major histocompatibility complexes, because the traits controlled by these complexes are inherited in codominant fashion [68], [69]. For example, in the case of murine CMV (whose virulence is MHC dependent), the F1 generation clearly shows the resistant parental phenotype [70]. Here the segregation pattern observed in the case of PVM demonstrates, on the contrary, that the MHC-I et –II gene products involved directly in antigen presentation to T lymphocytes and thereby in the development of an adaptive immune response are not responsible for the resistance of SJL/J.

### From PVM to RSV

Results collected here suggest that the genetic determinism of resistance to the pneumonia virus of mice is polygenic and that adaptive immunity is not involved. They confirm the conclusions of association studies in humans, because among the candidate genes potentially involved in the severity of infantile RSV disease, the strongest association was found for genes involved in innate immunity [71], [72]. Furthermore, since PVM infection in mouse is such a reliable model of infantile respiratory disease due to RSV [38], [39], identification of the resistance mechanisms at work in strain SJL/J may contribute significantly to understanding the human disease. In particular, as SJL/J is resistant to PVM but susceptible to other viruses [41], it should provide a basis for identifying pneumovirus-specific mechanisms. The priority is to perform positional cloning on the basis of crosses between SJL/J and 129/Sv or between SJL/J and DBA/2, in order to inventory the candidate genes. This could lead, as in man, to identifying many quantitative traits loci whose causative effect is hard to establish. It is therefore advisable to restrict the global resistance phenotype to a few elementary and relevant phenotypes. The most efficient strategy might be to take advantage of the known immunological specificities of SJL/J [73], some of which happen to concern cell lineages considered to be radioresistant: NK cells [74], [75] and macrophages [76], [77], [78]. Along with the neutrophils, they both are among the immune cells featuring the early response after RSV infection [79]. Although our findings do not suggest a major contribution of neutrophils in restricting PVM infection and replication, this remains to be deeply investigated, especially because neutrophils' role in case of severe RSV disease is still unclear [80]. The intervention of NK cells seems excluded, since whole-body irradiation causes the level of these cells to drop drastically without affecting resistance to PMV and because their cytolytic activity is constitutively low in SJL/J and is not increased by interferons (IFN) [81], [82]. The macrophages of SJL/J, on the other hand, are characterized by a constitutive IRF-3 activation, which allows them to develop an IFN response more quickly [83]. As for the human RSV [84], both PVM nonstructural proteins 1 and 2 are IFN-I antagonists [85], [86]; the fast and elevated IFN response of SJL/J mice may thus confer a better resistance to PVM infection. In parallel, alveolar macrophages depletion prior to PVM infection significantly enhance lung viral load of Balb/c mice despite a paradoxical prolonged survival [87]. Because the precise role of alveolar macrophages in the pathogenesis of infections due to RSV remains to be clarified [88], [89], investigating the contribution of SJL/J macrophages in PVM infection might thus bring an additional clue.
